# Supplementary material for: Sulfheme formation during homocysteine S-oxygenation by catalase in cancers and neurodegenerative diseases
Source: Nat Commun. 2016 Nov 16;7:13386. doi: 10.1038/ncomms13386 (PMC5116089; doi:10.1038/ncomms13386)
Supplement: Supplementary Information — Supplementary Figures 1-12, Supplementary Methods and Supplementary References. [file ncomms13386-s1.pdf]

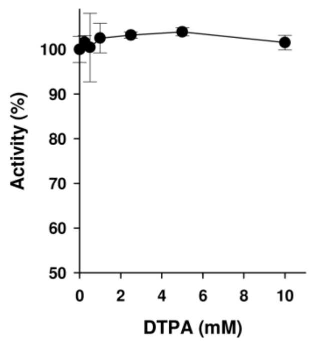

Supplementary Figure 1. DTPA does not interfere with the activity of catalase. Dependency of CAT activity on DTPA concentration at 25°C. CAT (40 nM) was pre-incubated for 120 min with DTPA (0-10 mM) before its activity was assayed as described in Methods. Data represent means ± s.d. of 3 independent experiments.

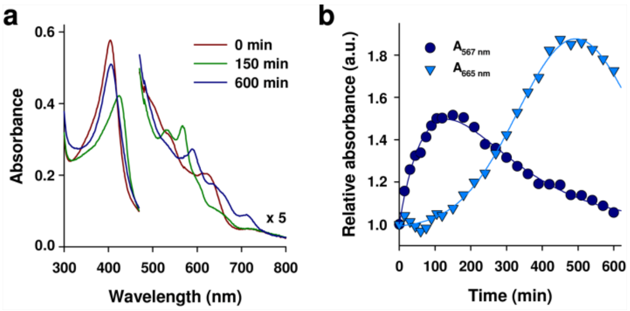

Supplementary Figure 2. HCys induces sulfheme formation under pathological conditions. (**a**) Representative experiment showing the UV-visible spectral changes recorded over time when catalase (1.8 μM) reacts with homocysteine (200 μM) in 50 mM KPi, pH 7.4 at 25°C. (**b**) Kinetics extracted from (**a**) at 567 nm (formation of compound II) and 665 nm (generation of the Fe(II) sulfonium species).

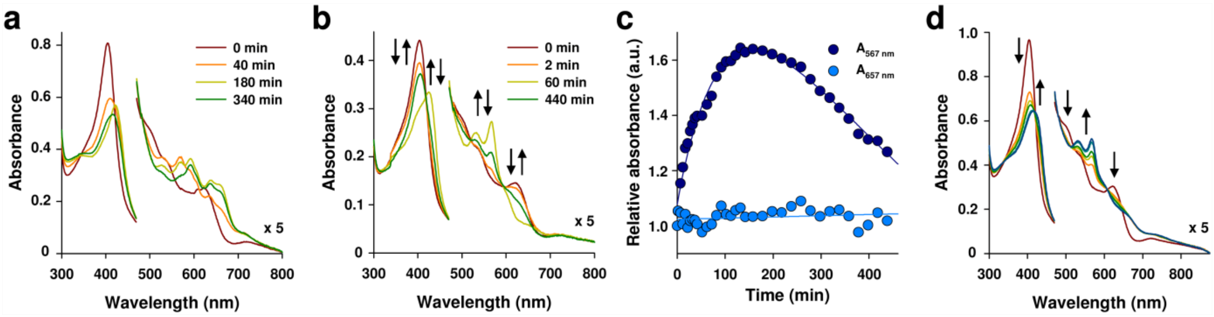

Supplementary Figure 3. Reactivity of catalase with Cys or GSH. (**a**) Representative experiments showing the spectral changes monitored by UV-visible spectroscopy during the reactivity of CAT (2.5 μM) with 9 mM Cys in 50 mM phosphate buffer, pH 7.4, 1 mM DTPA at 25°C. (**b**) Similar experiment than in (**a**) with CAT (1.35 μM) and Cys (200 μM) in the absence of DTPA. (**c**) Kinetics extracted from (**b**) at representative wavelengths, *i.e.* 567 nm (production of CAT-Fe(IV)=O) and 667 nm (generation of the Fe(II) sulfonium species). (**d**) Similar experiment than in (**a**) with CAT (2.95 μM) and GSH (30 mM).

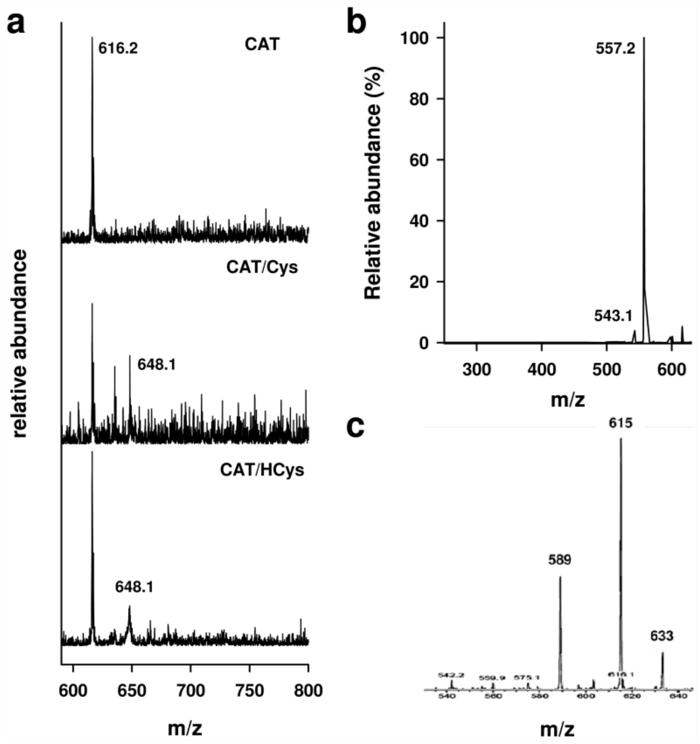

Supplementary Figure 4. Mass spectrometry analysis of sulfheme formation. (**a**) Mass spectra (ESI+) of the heme extracted from CAT (top), CAT incubated with 9 mM Cys (middle) or 2 mM HCys (bottom) for 8 hours in 50 mM phosphate buffer, pH 7.4, 1 mM DTPA at 25°C. (**b**) MS-MS spectrum (ESI+) of the molecular ion with a m/z = 616.2. The molecular ion at m/z = 543.1 and m/z = 557.2 result formally from the loss of a butyrate or from two decarboxylations, respectively. (**c**) MS-MS spectrum (ESI+) of the molecular ion with a m/z = 648.1. The molecular ion at m/z = 615.2 results formally from the concomitant loss of a •SH and the reduction of the heme-iron(III) in heme-iron(II).

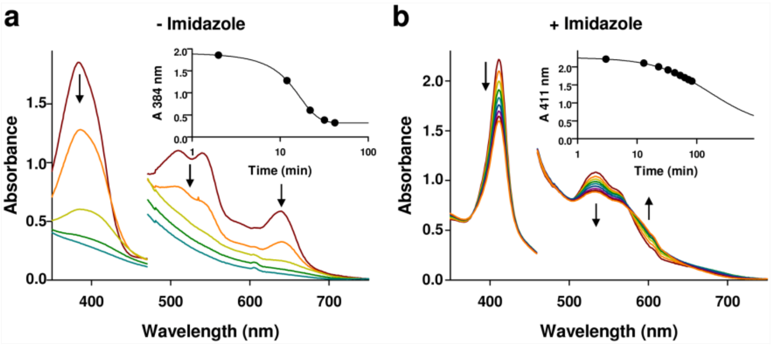

Supplementary Figure 5. Stabilization by imidazole of the heme-iron extracted from catalase. (**a**) UV-visible spectral changes recorded over time at 20°C of the heme-iron extracted from catalase with butan-2-one under acidic conditions. Inset: Time course of the decomposition of the extracted cofactor recorded at 384 nm. The data were fitted with a four parameter sigmoidal equation to extract the $t_{1/2}$. (**b**) Same experiment than in (**a**) in the presence of 2 M imidazole (10 % v/v). Inset: Time course of the stability of the extracted cofactor incubated with imidazole recorded at 411 nm. The data were fitted with a four parameter sigmoidal equation to extract the $t_{1/2}$ that are reported in the main text.

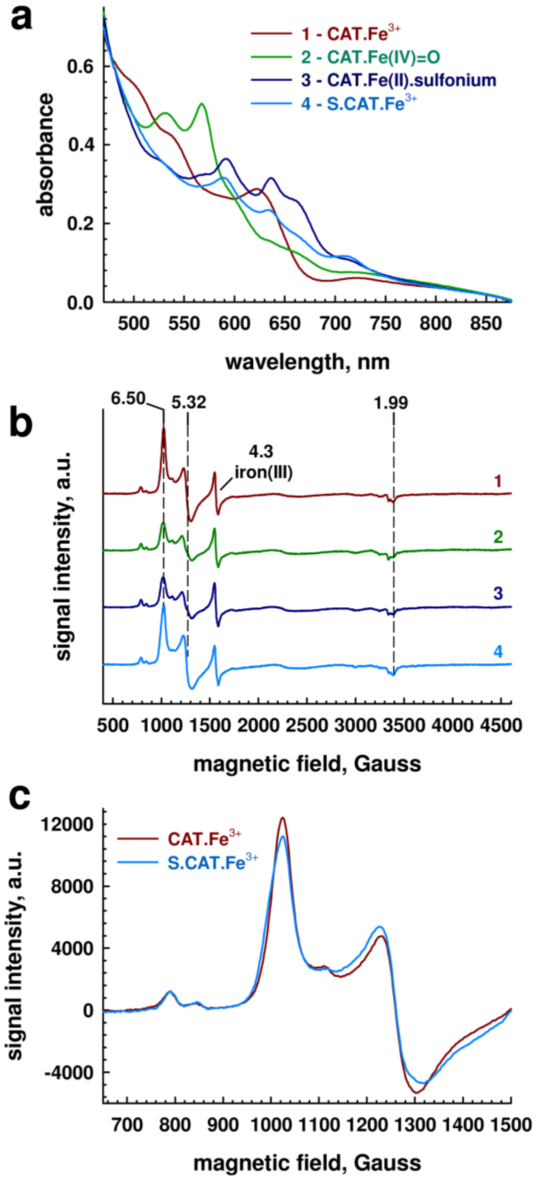

Supplementary Figure 6. EPR spectra recorded over time during the reactivity of CAT with HCys. (**a**) Spectral changes monitored by UV-visible spectroscopy over time during the reactivity of CAT (14.3 µM) with 8 mM HCys in 50 mM phosphate buffer, pH 7.4, 2 mM DTPA at 25°C. (**b**) X-band EPR spectra recorded at 10 K of the various species (28.6 µM CAT total) observed in (**a**). Settings were: modulation frequency, 100 kHz; modulation amplitude 1 mT; power, 20 mW. (**c**) Comparison of the High Spin EPR signal of native CAT-Fe(III) and the High Spin EPR signal resulting from the reactivity of CAT with HCys. Due to the half-site reactivity of CAT with HCys, the latter is a mixture of several High Spin species.

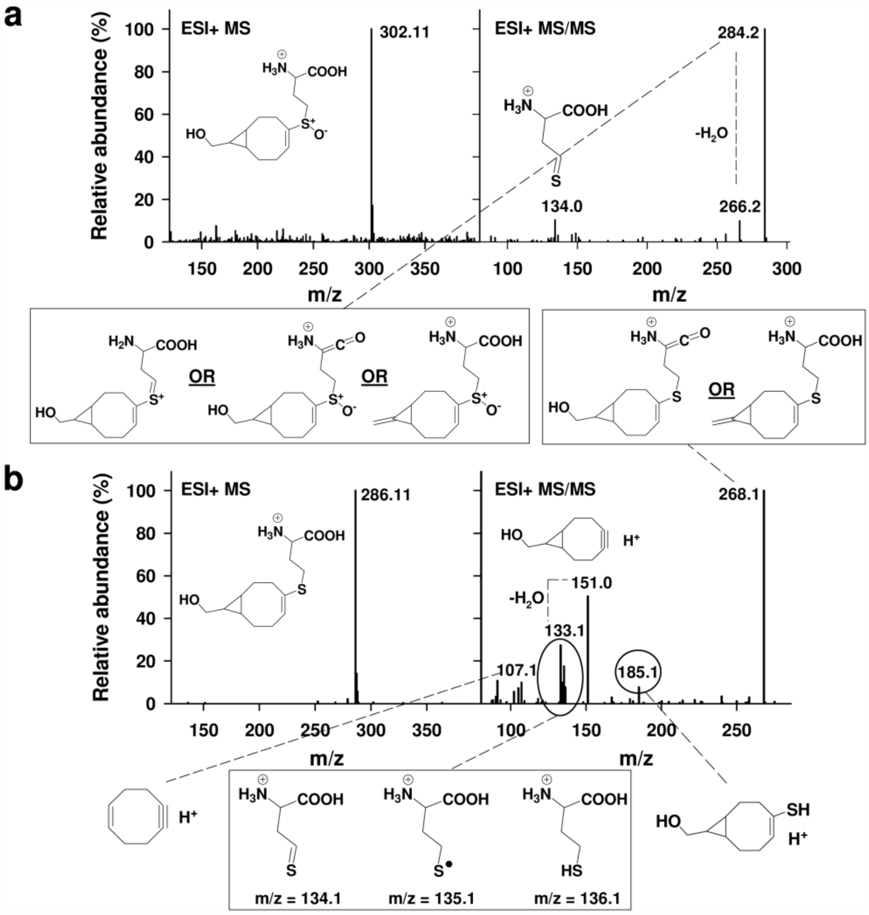

Supplementary Figure 7. The reactivity of CAT with HCys leads to the formation of thiyl radicals and sulfenic acid species. Mass spectrometry analyses of the BCN-HCys(S^+O^-) (**a**) and BCN-HCys (**b**) adducts observed during the reactivity of CAT (4 μM) with HCys (2 mM) in the presence of the bioconjugation agent bicyclo[6.1.0]nonyne (BCN, 0.1 mM) in 50 mM phosphate buffer, pH 7.4, 1 mM DTPA at 25°C.

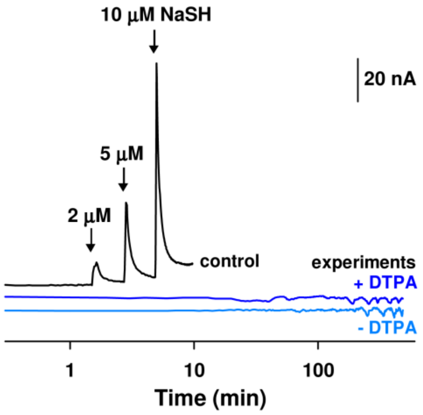

Supplementary Figure 8. Homocysteine-induced sulfheme formation takes place without H_2S intervention. Measurement for 8 hours of H_2S production with an ISO-H2S-2 Hydrogen Sulfide Sensor during the reactivity of CAT (3.4 μM) with HCys (2 mM) in 50 mM phosphate buffer, pH 7.4, 1 mM DTPA (experiment + DTPA) or with HCys (200 μM) in 50 mM phosphate buffer, pH 7.4 (experiment - DTPA). The calibration of the sensor (control) with a stock solution of NaSH (2-10 μM final) is shown for comparison.

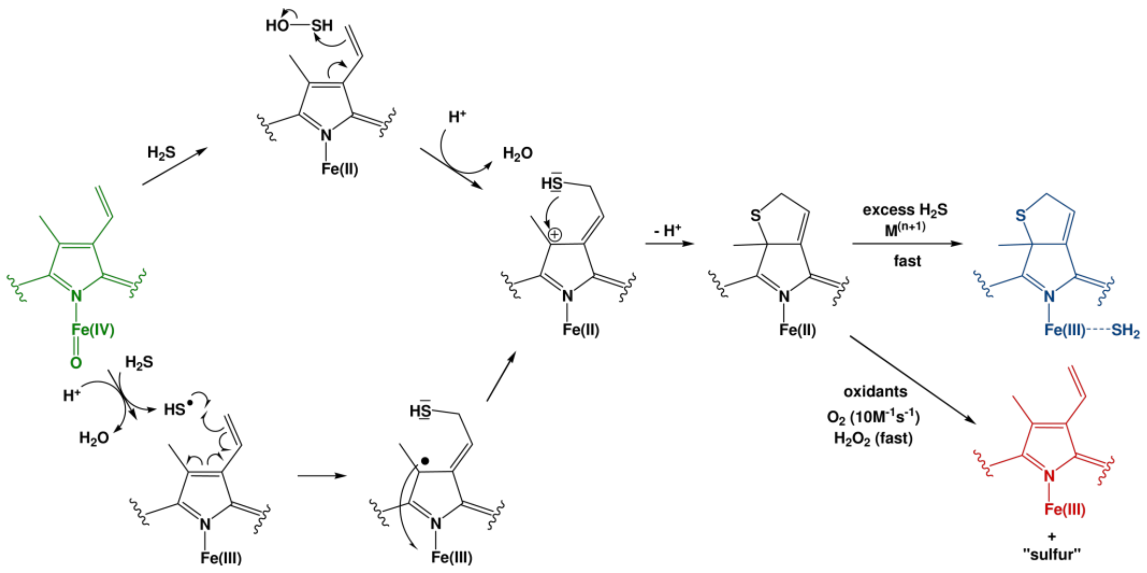

Supplementary Figure 9. Possible reaction mechanisms for the formation of Fe(II) sulfcatalase. The scheme depicts the two possible routes envisioned for the reactivity of an heme-iron(IV)-oxo species with H_2S to generate an iron(II) sulfheme species (left panel). The reactivity of CAT-Fe(IV)=O with H_2S takes place either through the reduction of the former by the latter (HS^-, H^+) to produce CAT-Fe(III) along with a sulfhydryl S•- radical or *via* the direct O atom transfer from the heme-iron(IV)-oxo species to H_2S to generate CAT-Fe(II) and an oxadisulfane species HSOH. The reactivity of Fe(II) sulfcatalase with an excess of H_2S or with oxidants (H_2O_2 or O_2)[1] is also shown (right panel).

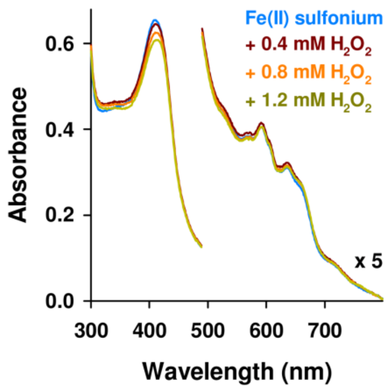

Supplementary Figure 10. Stability of the Fe(II) sulfonium species toward pathological concentrations of H_2O_2. Spectral changes monitored by UV-visible spectroscopy of the Fe(II) sulfonium species (2.85 μM) incubated with various amount of H_2O_2 in 50 mM phosphate buffer, pH 7.4, 1 mM DTPA at 25°C.

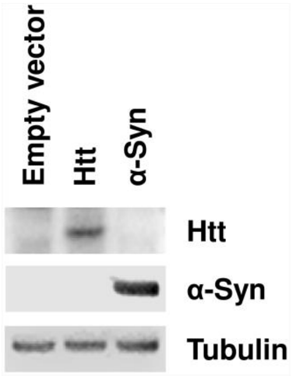

Supplementary Figure 11. Transfected Hek 293T cells express Htt or α-Syn. Hek293T cells were transfected with pcDNA (empty vector), Htt-N171-82Q (Htt) or Alpha-synuclein-A53T (α-Syn) plasmids (20µg) with the calcium phosphate method and the cells were harvested 48h after transfection. The expression of Htt or α-Syn in Hek 293T transfected cells was confirmed by Western blot analyses performed on cell lysates as described in Methods.

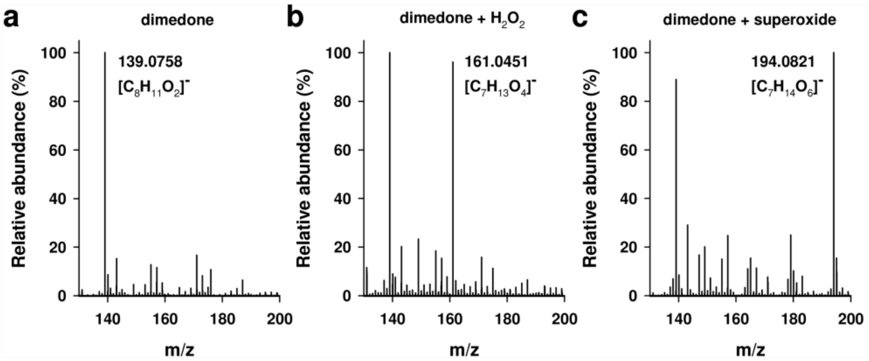

Supplementary Figure 12. Dimedone reacts with H_2O_2 and superoxide radical anions. High resolution mass spectra (ESI$^-$) of 100 μM dimedone (**a**) reacted with 1 equivalent H_2O_2 (**b**) or with $O_2\bullet^-$ generated by the enzymatic system xanthine (0.5 mM):xanthine oxidase (4 U) in the presence of 40 U CAT (**c**). For the latter, the reaction mixture was incubated for 30 min in 50 mM phosphate buffer, pH 7.4, 1 mM DTPA and then treated with acetic acid (20 % v/v). The reaction products were isolated on an Oasis® HLB cartridge (Waters) washed with distilled water and eluted with methanol.

SUPPLEMENTARY METHODS

EPR spectroscopy. EPR measurements were performed on a Bruker Elexsys 500 EPR spectrometer (Bruker, Wissembourg, France) operating at X-band (9.85 GHz) and equipped with a SHQ high-sensitivity cavity. For EPR measurements performed at 10 K, the spectrometer was equipped with a cryothermostat. For EPR spin trap experiments, typical settings used were microwave power, 20 mW; modulation frequency, 100 kHz; modulation amplitude, 0.1 mT; receiver gain, 60 dB; time constant, 40.96 ms; conversion time, 41.04 ms; data points, 1024; sweep width, 15 mT; sweep time, 42.02 s. EPR spectra were recorded sequentially during the whole reaction course at 21 °C. Typical experiments were conducted with a solution of CAT (50-200 μM) incubated with L-Cys or L-HCys (2 or 10 mM) or GSH (35 mM) and 200 mM DMPO in 50 mM KPi, pH 7.4, 1 mM DTPA. Control experiments (biological thiols alone) were also conducted. For EPR experiments at 10 K, typical settings were microwave power, 10 mW; modulation frequency, 100 kHz; modulation amplitude, 1 mT; receiver gain, 60 dB. Typical experiments were conducted with a solution of CAT (28-40 μM) incubated with 8-10 mM HCys in 50 mM KPi at pH 7.4, 2 mM DTPA at 25 °C. Spectral changes were followed by UV-visible spectrophotometry and after a desired time, aliquots were taken for EPR analysis. Data acquisition and processing were performed using Bruker Xepr software.

SUPPLEMENTARY REFERENCES

(1) Nicholls P. The formation and properties of sulphmyoglobin and sulphcatalase. *Biochem J.* **81,** 374-383 (1961).
